# Accelerated osteoarthritis in women with polycystic ovary syndrome: a prospective nationwide registry-based cohort study

**DOI:** 10.1186/s13075-021-02604-w

**Published:** 2021-08-30

**Authors:** Stefan Kluzek, Katrine Hass Rubin, Maria Sanchez-Santos, Mary S. O’Hanlon, Marianne Andersen, Dorte Glintborg, Bo Abrahamsen

**Affiliations:** 1 Academic Department of Orthopaedics, Trauma and Sports Medicine, Versus Arthritis – Centre for Sport, Exercise and Osteoarthritis Research, Faculty of Medicine and Health Sciences, University of Nottingham, Queens Medical Centre, Nottingham, NG7 2UH UK; 2 University of Southern Denmark, Odense, Denmark; 3 NDORMS, University of Oxford, Oxford, UK; 4 University of Nottingham, Nottingham, UK; 5 Odense University Hospital, Odense, Denmark; 6 Holbæk Hospital and University of Southern Denmark, Odense, Denmark

**Keywords:** Osteoarthritis, Polycystic ovary syndrome, Metabolic syndrome

## Abstract

**Background:**

Osteoarthritis (OA) is the most common form of arthritis with multiple risk factors implicated including female sex and obesity. Metabolic dysregulation associated with obesity leading to metabolic syndrome is a proposed component of that association. Polycystic ovary syndrome (PCOS) commonly affects women of reproductive age and these women are at higher risk of developing metabolic syndrome and thus likely to represent a high-risk group for early OA development. There are no published studies exploring the epidemiology of knee, hip and hand OA in women diagnosed with PCOS.

**Study aim:**

To assess the prevalence and incidence of knee, hip and hand osteoarthritis (OA) in women with polycystic ovary syndrome (PCOS) when compared with age-matched controls.

**Methods:**

Prospective Danish national registry-based cohort study. The prevalence of OA in 2015 and incidence rates of OA over 11.1 years were calculated and compared in more than 75,000 Danish women with either a documented diagnosis of PCOS ± hirsutism (during the period of 1995 to 2012) or age-matched females without those diagnoses randomly drawn from the same population register.

**Results:**

In 2015, the prevalence of hospital treated knee, hip and hand OA was 5.2% in women with PCOS diagnosis. It was 73% higher than that seen in age-matched controls. Significantly higher incidence rates were observed in the PCOS cohort compared with the age-matched controls during the follow-up period (up to 20 years), with the following hazard ratios (HR): 1.9 (95% CI 1.7 to 2.1) for knee, 1.8 (95% CI 1.3–2.4) for hand and 1.3 (95% CI 1.1 to 1.6) for hip OA. After excluding women with obesity, similar associations were observed for knee and hand OA. However, risk of developing hip OA was no longer significant.

**Conclusions:**

In this large prospective study, women with PCOS diagnosis had higher prevalence and accelerated onset of OA of both weight and non-weight bearing joints, when compared with age-matched controls. Further studies are needed to understand the relative effect of metabolic and hormonal changes linked with PCOS and their role in promoting development of OA.

**Supplementary Information:**

The online version contains supplementary material available at 10.1186/s13075-021-02604-w.

## Background

Osteoarthritis (OA) has the highest prevalence and incidence rates of all forms of arthritis. It is also expected to rise with increasing obesity and ageing population worldwide [1]. Osteoarthritis is associated with one of the most significant reductions of health-related quality of life in general population, and it has a big impact on health costs worldwide [2–4].

Several risk factors have been found to be associated with development of OA, including female sex, manual work and obesity [5–11]. The association between elevated body mass index (BMI) and OA is more significant for risk of developing knee and hand OA when compared with hip OA [12–15]. However, considering that obesity increases risk of OA in both non-weight-bearing and weight-bearing joints, excessive loading is unlikely to fully explain these associations. Presence of hand OA has also been shown to be an independent predictor for the future development of knee OA (KOA) even after adjustment for body mass index (BMI) [7, 16]. The relationship between obesity, hand OA and KOA implies at least a partial systemic or genetic component in the association between high BMI and OA.

Metabolic dysregulation associated with obesity can affect tissue homeostasis and has been proposed to be important component of that association. It is often diagnosed using metabolic syndrome (MetS) criteria. Although MetS has several specific definitions, it can be described as a combination of hypertension, dyslipidaemia, insulin resistance and abdominal obesity [17]. Individuals with OA have an increased prevalence of MetS [18] and individual components of MetS have also been associated with symptomatic OA in prospective studies in middle-age and elderly population [10, 19–25]. In addition, development of both OA and MetS has been associated not only with high BMI but also hormonal changes [8, 21, 26, 27].

Women have an increased risk of developing OA when compared with men, and it is particularly prevalent among post-menopausal women. These significant sex-related differences in incidence after menopause would suggest that sudden changes in sex steroid levels play a role in OA pathogenesis [28, 29]. In younger females, increasing oestradiol levels, higher BMI and elevated blood pressure are also factors associated with a greater risk of OA, while higher levels of testosterone potentially have a mildly protective role [30]. On the other hand, some radiographic studies have suggested oestrogen hormone replacement therapy (HRT) use may also have a protective effect of on the radiographic detection of OA or its progression in older population of women [31–35].

Polycystic ovary syndrome (PCOS) is the most common endocrine disorder in women of reproductive age [36]. Signs of hyperandrogenism are part of the diagnostic process, and the majority of women with hirsutism are found to have PCOS [37, 38]. Women with PCOS are at a higher risk of developing the individual components of MetS, including central obesity, insulin resistance, dyslipidaemia and hypertension [39–41] than the general population, and these components are frequently diagnosed earlier, at a pre-menopausal age. These metabolic changes could predispose to accelerated development of OA.

It is currently not known if women with PCOS are a high-risk group for accelerated development of OA. Although estimates of incident OA have been recorded from other populations, there are no published studies specifically exploring the epidemiology of knee, hip and hand OA in women diagnosed with PCOS [7]. Contrary to the effects of the components of MetS, the androgen excess often observed in individuals with PCOS may be associated with a decreased risk of developing OA.

Therefore, the main objectives of this study were to assess and compare the prevalence and incidence rates of clinically diagnosed knee, hip and hand OA among women with and without PCOS in a large cohort from the Danish National Patient Register (NPR).

## Methods

This is a register-based study including two cohorts, one with PCOS patients and a control population. Patients with PCOS were identified from the Danish National Patient Register according to the International Classification of Diseases (ICD) 10 with the diagnoses of hirsutism (L68.0) and/or PCOS (E28.2) [39, 42]. The inclusion of hirsutism as an identifier of PCOS reflects the varying PCOS defining criteria used in the register during the study time period, particularly before the Rotterdam criteria were introduced in 2003 [36, 39]. Furthermore, the majority of women (> 80%) with clinical hyperandrogenism were found to have PCOS in earlier studies [37–39].

The study was approved by the Data Protection Agency and by the Statistics Denmark (project no 704175).

### Study population

**Fig. 1:**
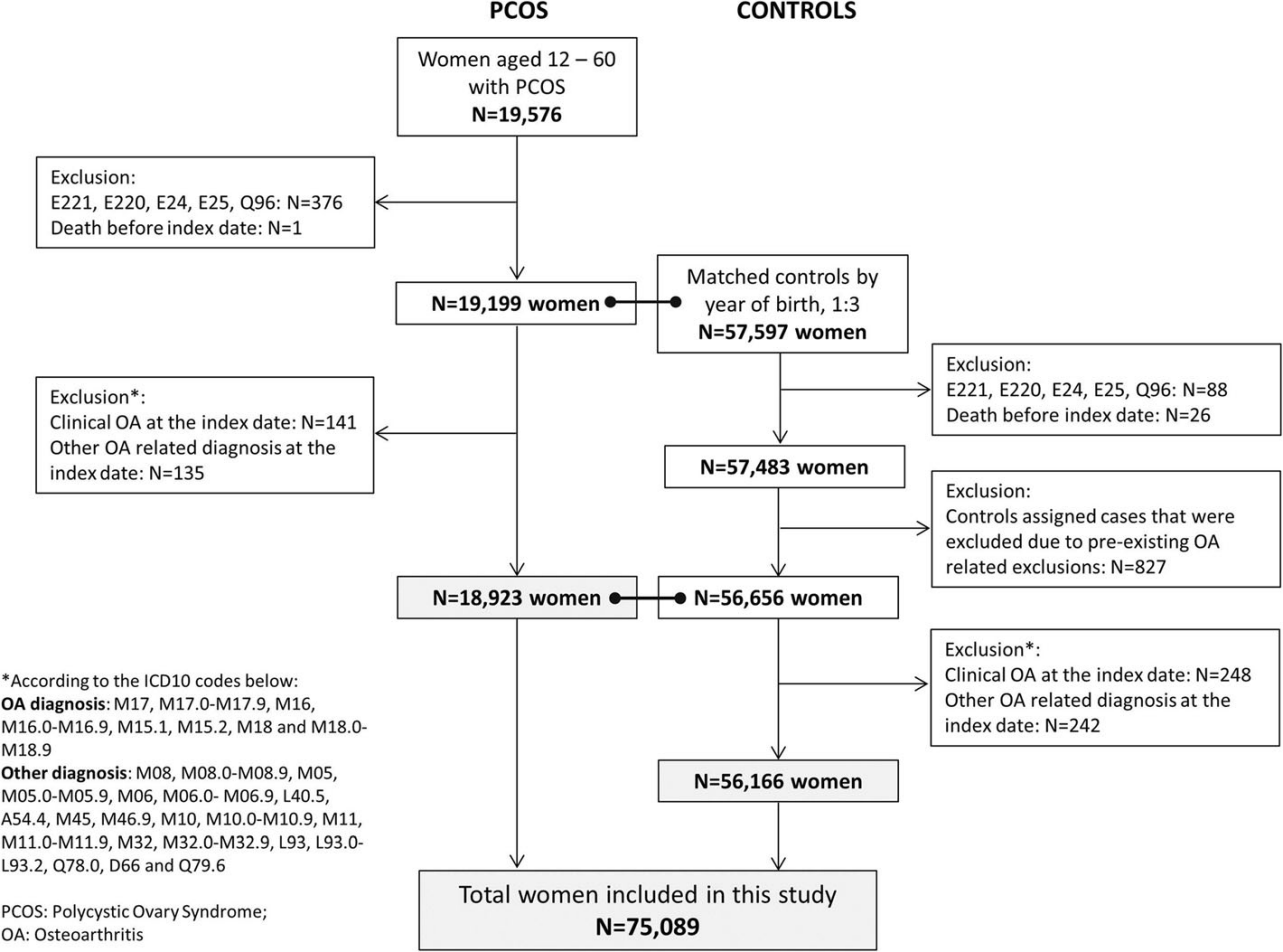
Flowchart explaining the selection of individuals listed in the Danish National Patient Register for inclusion in the study cohorts

The study design and baseline data for this cohort have recently been reported in detail [39]. In brief, Danish women aged 12 years to 60 years with a documented diagnosis of PCOS and/or hirsutism during the period of 1995 to 2012 were included as cases in this study. The index date was the first date of PCOS diagnosis. For each PCOS case, three controls were randomly drawn from the civil population register. Selection criteria for the controls were female sex and same year of birth as each PCOS case and alive on the index date of their respective PCOS case (Fig. 1). The distribution of demographic and clinical characteristics in PCOS patients and controls are shown in Tables 1 and 2.

**Table 1:** Characteristics of PCOS patients and controls

		PCOS		Controls		
		*N* = 18,923		*N* = 56,166		
		*N*	%	*N*	%	*p* value
Age (years) at first diagnosis, median (Q1–Q3)		29 (24–36)		29 (24-36)		0.523
< 30 years		9592 (51%)		28,621 (51%)		0.985
30 to < 35 years		3756 (20%)		11,160 (20%)		
35 to < 40 years		2497 (13%)		7394 (13%)		
40 to < 45 years		1366 (7%)		4023 (7%)		
45 to < 50 years		848 (4%)		2476 (4%)		
50 to < 55 years		515 (3%)		1496 (3%)		
55 to < 60 years		349 (2%)		996 (2%)		
Comorbidity (Charlson index ≥1)		1204	6.36	2.236	3.98	< 0.001
**Cardiometabolic diseases**	**ICD-10 codes**					
Obesity	E66	2263	11.96	759	1.35	< 0.001
Diabetes	E10 to E14	414	2.19	407	0.72	< 0.001
Type 1 diabetes	E10	159	0.84	298	0.53	< 0.001
Type 2 diabetes	E11	294	1.55	167	0.30	< 0.001
Gestational diabetes	O24, P70	285	1.51	211	0.38	< 0.001
Dyslipidaemia	E78	101	0.53	100	0.18	< 0.001
Hypertension	I109	318	1.68	331	0.59	< 0.001
**Cardiovascular disease**	I20, I50	67	0.35	161	0.29	0.145
Myocardial infarction	I21 to I25	34	0.18	114	0.20	0.532
Transient cerebral ischemia	G45	25	0.13	46	0.08	0.052
Stroke	I63, I64	45	0.24	75	0.23	0.002
Thrombosis, lung embolism	I26, O082, I80 to I82	121	0.64	168	0.30	< 0.001

*ICD* International Classification of Diseases

**Table 2:** Incidence rates per 1000 person year (PY) for knee, hip and hand osteoarthritis between patients with and without PCOS

	PCOS		Controls		Unadjusted HR^a^ (95% CI)	*p* value
	No. of events	Incidence rate per 1,000 PY (95%CI)	No. of events	Incidence rate per 1000 PY (95% CI)		
*Knee osteoarthritis*	588	2.8 (2.5; 3.0)	951	1.5 (1.4; 1.6)	1.9 (1.7;2.1)	< 0.001
*Hip osteoarthritis*	173	0.8 (0.7; 0.9)	390	0.6 (0.6; 0.7)	1.3 (1.1; 1.6)	0.004
*Hand osteoarthritis*	55	0.3 (0.2; 0.3)	96	0.1 (0.1; 0.2)	1.8 (1.3; 2.4)	0.001

^a^Reference = control group

*PCOS* polycystic ovary syndrome, *HR* hazard ratio

### Exclusion criteria

1. For the estimation of prevalence at the end of the study, individuals with a presence of the following conditions were excluded: juvenile arthritis, rheumatoid arthritis (RA), psoriatic arthritis, spondyloarthropathy, gout, other crystal arthropathies, lupus and osteogenesis imperfecta, Ehlers-Danlos and symptomatic haemophilia (Fig. 1). The ICD-10 codes used to ascertain these diagnoses are shown in supplementary Table S1.
2. For the incidence rate analyses, further criteria were applied: women with the presence of OA at the index date (from the first time a patient received the PCOS diagnosis, and with an index date assigned for the controls) were excluded (Fig. 1).

### Outcome

The outcome was clinically diagnosed knee, hip and hand OA according to the ICD-10 codes.

Clinical diagnoses of incident knee, hip and hand OA took place during the study period from index date to end of study (December 31, 2015). ICD-10 codes to identify these diagnoses have been previously validated elsewhere [7] and are shown in Supplementary Table S1.

### Comorbidities

Cardiometabolic comorbidities, the Charlson Comorbidity Index and major adverse cardiac events preceding the index date presented in this study have been previously used elsewhere [39].

### Statistical analysis

Data analysis was performed using Stata software version 15 (Stata Corp LP., College Station, TX). Descriptive analysis of baseline characteristics preceding the first diagnosis of PCOS of the overall population and by age groups was conducted. Categorical variables were presented as relative and absolute frequencies and continuous variables as the mean ± SD or median (25–75 percentile), depending on the nature of their distribution. For the comparison of categorical variables between the sexes, Pearson’s *χ*^2^ test was used and Fisher’s exact test when at least 20% of the cells had expected frequencies less than five. Prevalence was calculated by dividing the total numbers of patients with OA by the number of eligible individuals in the registry as the denominator representing 73,561 women in 2015.

Incidence rates of clinical OA were estimated in patients with and without PCOS (and without presence of OA at the index date), both overall and per age categories. They were calculated as the number of new cases of OA over the total person time at risk. The person time at risk for each woman was estimated as the time each woman remains free of OA during the follow-up period (up to 20 years). Hazard ratio (HR) and 95% confident of intervals (CIs) were assessed to quantify the magnitude of the association between patients with and without PCOS and the incidence of OA. Cox proportional hazard regression was used. Women who did not develop OA during follow-up were right-censored at the earliest of loss to follow-up, end of the study period or death. Linearity and proportionality of the hazards were checked.

One sensitivity analysis was conducted to overview role of obesity as a main risk factor for weight bearing and non-weight bearing joint OA. Therefore, women with a hospital history of obesity (ICD-10 code E66) before the index date were excluded in this sensitivity analysis.

## Results

For the analysis of prevalence in 2015, data were available for 75,088 women: 18,844 with PCOS and 56,244 controls. The prevalence of hospital treated knee, hip and hand OA that year was 5.2%, and it was significantly higher in population with PCOS when compared with age-matched controls. The most prevalent joint OA in PCOS cohort was in the knee (3.7%), followed by the hip (1.1%) and then the hand (0.4%) (see Supplementary Table S2). As expected, the most constant trend of observed prevalence ratios across all age groups was for knee OA (see Supplementary Figure S1).

For the calculation of incidence, from the index day until last follow-up, data were available in 75,089 women: 18,923 (25.2%) with PCOS and 56,166 (74.8%) controls. The median age was 29 years at the time of index. During the observation period (1995 to 2015), 2253 women had a clinical diagnosis of incident knee, hand or hip OA, of whom 816 (4.3%) were patients from the PCOS cohort and 1437 (2.6%) were from the control group. Significantly higher incidence rates per 1000 person years were observed in the PCOS cohort when compared with the control group in the three different joints (2.8 vs. 1.5, 0.3 vs. 0.1 and 0.8 vs. 0.6 for knee, hand and hip, respectively). Overall, the PCOS cohort had a higher HR for developing knee, hand and hip OA. The risk of developing OA was 90% higher (HR 1.9, 95% CI 1.7 to 2.1) for the knee, 80% (HR 1.8, 95% CI 1.3–2.4) for the hand and 30% (HR 1.3, 95% CI 1.1 to 1.6) for the hip in the PCOS cohort compared to the control (Table 2). Women at the age of 50 to 60 were more likely to develop OA that those who were younger than 45 years old. Age stratified analyses for KOA were significant for all age groups in the knee analyses but not in the hand and hip analyses (Tables S3-S5).

**Fig. 2:**
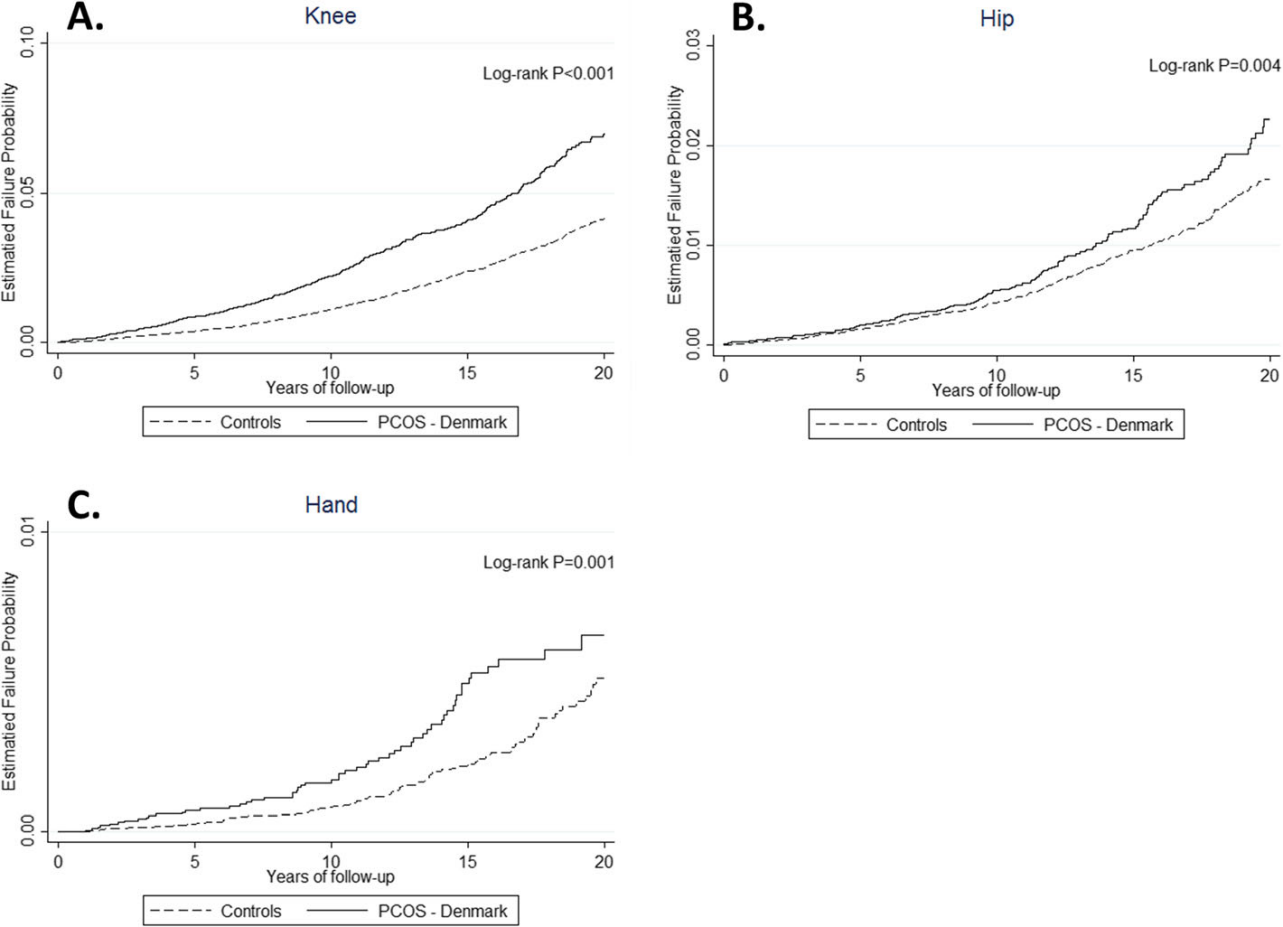
Incidence of osteoarthritis in PCOS and control cohorts. Kaplan-Meier failure plots of osteoarthritis incidence up to 20 years for knee (**A**), hip (**B**) and hand (**C**) osteoarthritis

After excluding women with obesity, similar results were observed with the higher risk of knee (HR 1.6, 95% CI 1.4 to 1.8) and hand OA (HR 1.6, 95% CI 1.1 to 2.3) in the PCOS cohort. However, risk of developing hip OA (HR 1.2, 95% CI 1.0 to 1.5) was no longer significant after women diagnosed with obesity were excluded from the analysis (Tables S3-S5). Kaplan–Meier failure curves for incidence of knee, hip and hand OA are presented in Fig. 2. Women with PCOS had a greater risk of all examined joints OA when compared with women without PCOS (log-rank test *p* ≤ 0.001).

As expected and previously reported, all components of metabolic syndrome were more common in PCOS than control cohort [39]. Furthermore, the rates of cardiometabolic comorbidities, the Charlson Comorbidity Index and major adverse cardiac events at the index date were compared between PCOS cases and controls and presented in Table 1.

## Discussion

In this prospective cohort study, we found that women with PCOS—the most common endocrine disorder in young and middle-aged females—have both a higher prevalence and higher risk of developing OA requiring hospital treatment compared with age-matched controls from the same population. The association between PCOS and incidence of OA was particularly strong for KOA and significant in all age groups, including women below the age of 45. The HR was similar for the knee and hand OA, but significantly weaker for hip OA. No substantial differences in the results were observed when obese women were excluded from the analysis. Furthermore, considering that we observed an increased incidence of OA in both weight-bearing and non-weight-bearing joints, excessive loading associated with higher body weight alone is unlikely to explain the accelerated development of OA in women with PCOS.

The potentially protective effect of androgen excess for OA development in premenopausal women [30], which is characteristic for patients with PCOS, was not observed. As expected, individuals with PCOS had a higher prevalence of cardiometabolic diseases and obesity when compared with age-matched controls from the same population [39]. However, the risk of developing OA for women with PCOS could be discrepant between those who had higher BMI and those who had androgen excess in the presence of a normal body weight.

This is the first study, to our knowledge, to describe the association between PCOS and the accelerated risk of developing OA in both weight-bearing and non-weight-bearing joints. There are number of plausible explanations for our findings, which are not mutually exclusive. The most biologically plausible mechanism for the impact of PCOS on increased OA risk is the higher BMI due to excess adipose tissue in the PCOS population when compared with controls. In OA, this has been attributed to excessive loading and to the impact of metabolic dysregulation and systemic inflammation on the regeneration and remodelling of joint tissues. The relative contribution of excessive adipose tissue on biomechanical and metabolic changes affecting each joint is unknown. It is difficult to disentangle the relative importance of these intercorrelated risk factors for developing OA [43, 44]. Metabolic and hormonal changes are likely to influence tissue-remodelling processes accelerated by the direct effect of excessive mechanical loading. Visser et al. [45] found in a large population-based cross-sectional study, with an oversampling of persons with high BMI, that clinically diagnosed knee OA was mainly associated with surrogate markers for mechanical stress, whereas hand OA with MetS. However, Monira Hussain et al. showed in a large prospective cohort study that MetS was associated with increased risk of severe knee OA (requiring knee replacement) independent of BMI. Furthermore, that study also showed no such relationship for severe hip OA [25].

Interestingly, for non-weight bearing joints, our study supports the hypothesis that even young individuals with metabolic aberrations accompanied by increased low-grade inflammation, observed in PCOS, are at higher risk of developing OA [46] requiring referral to Danish hospital clinics. Accordingly, differences between knee, hand and hip OA in the PCOS group mimic the strength of association between knee, hand and hip OA and increased risk of early mortality [47–49].

### Strengths and limitations

Participants’ selection to enter this study was not limited to specific health centres and included all those with a PCOS and/or hirsutism diagnosis available on the Danish NPR. The comparison group was part of the same population and both groups had no OA at baseline.

Some potential limitations of this study are worth mentioning. Findings are derived from consults at Danish hospital clinics, hence focused on more severe cases of OA than in Primary Care. Health care seeking behaviour of this population, which might affect both incidence rates of OA and diagnosis of PCOS and/or hirsutism, might be different to other health care systems in other parts of the world. However, universal access to health care is the underlying principle inscribed in Denmark’s Health Law, with life expectancy and health outcomes in Denmark similar to the EU average [50]. As this is the first study describing prevalence and incidence of OA in PCOS population, we are not able to compare it with previous results from other countries. However, the Danish population has similar rates of common diseases to other European states [51–53]. Therefore, we see no reason to assume that those results are different in other countries.

The potential bias could be associated with surveillance bias, which has been shown to be higher for those with a diagnosis of chronic illness. The greater observational intensity usually leads to healthier people being labelled as chronically ill and as a result of additional diagnostic tests and treatments lead to better long-term outcomes when compared with the sex and age matched population. However, observational intensity bias seems to be unlikely for the present study, where with the examined women with PCOS being shown to have substantially worse outcomes in terms of mortality when compared with the matched population [39, 54].

PCOS incorporates symptoms related to the reproductive system and systemic metabolic abnormalities associated with an increased risk for cardiovascular disease. Previous studies have linked PCOS with MetS and excessive cardiovascular problems. However, MetS and OA share the same risk factor: high BMI. Despite comprehensive baseline data regarding obesity and cardiometabolic comorbidities, we did not adjust our analysis as those factors represent potential components of MetS and would represent intermediate variables on a causal path between exposure and outcome and as such represent over-adjustment bias [55]. For the same reason, we did not examine the factors associated with PCOS, such as elevated coagulation factors or adipokine levels, which can contribute to increased OA and cardiovascular risk. The NPR does not contain accurate information regarding BMI measurements but only codes associated with a significant obesity. Therefore, individuals with milder degrees of obesity could be potentially misclassified as non-obese.

## Conclusions

In conclusion, in 2015, prevalence of hospital treated knee, hip and hand OA was higher in Danish population with PCOS when compared with age-matched controls. Furthermore, a PCOS diagnosis was associated with accelerated development of OA when compared with age-matched controls from the same population and this was particularly strong for knee and hand OA.

The link behind this relationship is not completely understood and further research analysing the specific role of MetS components is required to identify potential underlying mechanisms. Assessing the effect of the common treatments used in PCOS on the prevention of OA could help develop new OA treatments in the near future [56]. In the first instance, this will require an understanding of the relative risk of OA for the specific phenotypes of PCOS. The potentially protective effect of androgen excess in pre-menopausal women [30], which is characteristic for patients with PCOS [37], was either non-significant or overshadowed by the disadvantageous effect of metabolic dysregulation in this study.

## Supplementary Information

**Additional file 1: Table S1**. ICD-10 codes used for definition of exposure (PCOS), outcomes (hand, hip and knee clinical OA) and exclusion diagnosis. **Table S2**. Prevalence of knee, hip and hand osteoarthritis in 2015 in Danish population with clinical diagnosis of PCOS and match controls stratified by age groups. **Table S3**. Prevalence of knee, hip and hand osteoarthritis in 2015 in participants with PCOS, matched controls and combined population group stratified by outcomes (hand, hip and knee clinical OA). **Figure S1**. Prevalence ratios in 2015 between PCOS and control cohorts for knee, hip and hand osteoarthritis stratified by age groups. **Table S4**. Incidence rates per 1000 person-year (PY) for knee osteoarthritis by age groups (age at baseline) between patients with and without PCOS. **Table S5**. Incidence rates per 1000 person-year (PY) for hip osteoarthritis by age groups at baseline between patients with and without PCOS. **Table S6**. Incidence rates per 1000 person-year (PY) for hand osteoarthritis by age groups at baseline between patients with and without PCOS.

## Data Availability

The datasets generated and/or analysed during the current study are available in the Danish National Patient Registry (DNPR) repository https://econ.au.dk/the-national-centre-for-register-based-research/danish-registers/the-national-patient-register/.

## Abbreviations

BMI: Body mass index
CI: Confidence interval
DNPR: Danish National Patient Register
HR: Hazard ratio
HRT: Hormone replacement therapy
ICD: International Classification of Diseases
MetS: Metabolic syndrome
OA: Osteoarthritis
PY: Per year
PCOS: Polycystic ovary syndrome
RA: Rheumatoid arthritis

## References

[1] Vina ER, Kwoh CK. Epidemiology of osteoarthritis: literature update. Curr Opin Rheumatol. 2017. 10.1097/BOR.0000000000000479 PMC5832048 29227353

[2] Salmon JH, Rat AC, Sellam J, Michel M, Eschard JP, Guillemin F, Jolly D, Fautrel B (2016). Economic impact of lower-limb osteoarthritis worldwide: a systematic review of cost-of-illness studies. Osteoarthr Cartil.

[3] Wu M, Brazier JE, Kearns B, Relton C, Smith C, Cooper CL (2015). Examining the impact of 11 long-standing health conditions on health-related quality of life using the EQ-5D in a general population sample. Eur J Health Econ.

[4] March L, Smith EU, Hoy DG, Cross MJ, Sanchez-Riera L, Blyth F (2014). Burden of disability due to musculoskeletal (MSK) disorders. Best Pract Res Clin Rheumatol.

[5] Bierma-Zeinstra SM, Koes BW (2007). Risk factors and prognostic factors of hip and knee osteoarthritis. Nat Clin Pract Rheumatol.

[6] Lievense AM, Bierma-Zeinstra SM, Verhagen AP, van Baar ME, Verhaar JA, Koes BW (2002). Influence of obesity on the development of osteoarthritis of the hip: a systematic review. Rheumatology (Oxford, England).

[7] Prieto-Alhambra D, Judge A, Javaid MK, Cooper C, Diez-Perez A, Arden NK (2014). Incidence and risk factors for clinically diagnosed knee, hip and hand osteoarthritis: influences of age, gender and osteoarthritis affecting other joints. Ann Rheum Dis.

[8] Reyes C, Leyland KM, Peat G, Cooper C, Arden NK, Prieto-Alhambra D (2016). Association between overweight and obesity and risk of clinically diagnosed knee, hip, and hand osteoarthritis: a population-based cohort study. Arthritis Rheumatol (Hoboken, NJ).

[9] Kluzek S, Newton JL, Arden NK (2015). Is osteoarthritis a metabolic disorder?. Br Med Bull.

[10] Wang H, Cheng Y, Shao D, Chen J, Sang Y, Gui T (2016). Metabolic syndrome increases the risk for knee osteoarthritis: a meta-analysis. Evid Based Complement Alternative Med.

[11] Yucesoy B, Charles LE, Baker B, Burchfiel CM (2015). Occupational and genetic risk factors for osteoarthritis: a review. Work (Reading, Mass).

[12] Blagojevic M, Jinks C, Jeffery A, Jordan KP. Risk factors for onset of osteoarthritis of the knee in older adults: a systematic review and meta-analysis. Osteoarthr Cartil. 2010;18(1):24–33. 10.1016/j.joca.2009.08.010. Epub 2009 Sep 2. 10.1016/j.joca.2009.08.010 19751691

[13] Lee R, Kean WF (2012). Obesity and knee osteoarthritis. Inflammopharmacology..

[14] Grotle M, Hagen KB, Natvig B, Dahl F, Kvien TK (2008). Obesity and osteoarthritis in knee, hip and/or hand: an epidemiological study in the general population with 10 years follow-up. BMC Musculoskelet Disord.

[15] Yusuf E, Nelissen RG, Ioan-Facsinay A, Stojanovic-Susulic V, DeGroot J, van Osch G, Middeldorp S, Huizinga TWJ, Kloppenburg M (2010). Association between weight or body mass index and hand osteoarthritis: a systematic review. Ann Rheum Dis.

[16] Dahaghin S, Bierma-Zeinstra SMA, Reijman M, Pols HAP, Hazes JMW, Koes BW (2005). Does hand osteoarthritis predict future hip or knee osteoarthritis?. Arthritis Rheum.

[17] Levesque J, Lamarche B (2008). The metabolic syndrome: definitions, prevalence and management. J Nutrigenetics Nutrigenomics.

[18] Puenpatom RA, Victor TW (2009). Increased prevalence of metabolic syndrome in individuals with osteoarthritis: an analysis of NHANES III data. Postgrad Med.

[19] Zhang YM, Wang J, Liu XG (2017). Association between hypertension and risk of knee osteoarthritis: a meta-analysis of observational studies. Medicine..

[20] Zhang W, Randell EW, Sun G, Likhodii S, Liu M, Furey A, Zhai G (2017). Hyperglycemia-related advanced glycation end-products is associated with the altered phosphatidylcholine metabolism in osteoarthritis patients with diabetes. PLoS One.

[21] Niu J, Clancy M, Aliabadi P, Vasan R, Felson DT (2017). Metabolic syndrome, its components, and knee osteoarthritis: the Framingham Osteoarthritis Study. Arthritis Rheumatol (Hoboken, NJ).

[22] Lo GH, McAlindon TE, Katz JN, Driban JB, Price LL, Eaton CB (2017). Systolic and pulse pressure associate with incident knee osteoarthritis: data from the Osteoarthritis Initiative. Clin Rheumatol.

[23] Askari A, Ehrampoush E, Homayounfar R, Arasteh P, Naghizadeh MM, Yarahmadi M, Tarbiat N, Eghbali SS (2017). Relationship between metabolic syndrome and osteoarthritis: the Fasa Osteoarthritis Study. Diabetes Metab Syndrome.

[24] Williams MF, London DA, Husni EM, Navaneethan S, Kashyap SR (2016). Type 2 diabetes and osteoarthritis: a systematic review and meta-analysis. J Diabetes Complicat.

[25] Monira Hussain S, Wang Y, Cicuttini FM, Simpson JA, Giles GG, Graves S, Wluka AE (2014). Incidence of total knee and hip replacement for osteoarthritis in relation to the metabolic syndrome and its components: a prospective cohort study. Semin Arthritis Rheum.

[26] Grundy SM, Cleeman JI, Daniels SR, Donato KA, Eckel RH, Franklin BA, Gordon DJ, Krauss RM, Savage PJ, Smith SC, Spertus JA, Costa F (2005). Diagnosis and management of the metabolic syndrome. An American Heart Association/National Heart, Lung, and Blood Institute Scientific Statement. Executive summary. Cardiol Rev.

[27] Huffman KM, Kraus WE (2012). Osteoarthritis and the metabolic syndrome: more evidence that the etiology of OA is different in men and women. Osteoarthr Cartil.

[28] Fenton A, Panay N (2016). Estrogen, menopause and joints. Climacteric.

[29] Bay-Jensen AC, Slagboom E, Chen-An P, Alexandersen P, Qvist P, Christiansen C (2013). Role of hormones in cartilage and joint metabolism: understanding an unhealthy metabolic phenotype in osteoarthritis. Menopause (New York, NY).

[30] Sowers MF, Hochberg M, Crabbe JP, Muhich A, Crutchfield M, Updike S (1996). Association of bone mineral density and sex hormone levels with osteoarthritis of the hand and knee in premenopausal women. Am J Epidemiol.

[31] Hannan MT, Felson DT, Anderson JJ, Naimark A, Kannel WB (1990). Estrogen use and radiographic osteoarthritis of the knee in women. The Framingham Osteoarthritis Study. Arthritis Rheum.

[32] Hart DJ, Doyle DV, Spector TD (1999). Incidence and risk factors for radiographic knee osteoarthritis in middle-aged women: the Chingford Study. Arthritis Rheum.

[33] Nevitt MC, Cummings SR, Lane NE, Hochberg MC, Scott JC, Pressman AR, Genant HK, Cauley JA (1996). Association of estrogen replacement therapy with the risk of osteoarthritis of the hip in elderly white women. Study of Osteoporotic Fractures Research Group. Arch Intern Med.

[34] Spector TD, Nandra D, Hart DJ, Doyle DV (1997). Is hormone replacement therapy protective for hand and knee osteoarthritis in women?: The Chingford Study. Ann Rheum Dis.

[35] Zhang Y, McAlindon TE, Hannan MT, Chaisson CE, Klein R, Wilson PW (1998). Estrogen replacement therapy and worsening of radiographic knee osteoarthritis: the Framingham Study. Arthritis Rheum.

[36] Rotterdam EA-SPcwg (2004). Revised 2003 consensus on diagnostic criteria and long-term health risks related to polycystic ovary syndrome (PCOS). Hum Reprod.

[37] Glintborg D, Andersen M (2010). An update on the pathogenesis, inflammation, and metabolism in hirsutism and polycystic ovary syndrome. Gynecol Endocrinol.

[38] Azziz R (2003). The evaluation and management of hirsutism. Obstet Gynecol.

[39] Glintborg D, Hass Rubin K, Nybo M, Abrahamsen B, Andersen M (2015). Morbidity and medicine prescriptions in a nationwide Danish population of patients diagnosed with polycystic ovary syndrome. Eur J Endocrinol.

[40] Couto Alves A, Valcarcel B, Makinen VP, Morin-Papunen L, Sebert S, Kangas AJ (2017). Metabolic profiling of polycystic ovary syndrome reveals interactions with abdominal obesity. Int J Obes.

[41] Zhang Z, Hong Y, Chen M, Tan N, Liu S, Nie X, Zhou W (2020). Serum metabolomics reveals metabolic profiling for women with hyperandrogenism and insulin resistance in polycystic ovary syndrome. Metabolomics..

[42] Rubin KH, Glintborg D, Nybo M, Andersen M, Abrahamsen B (2016). Fracture risk is decreased in women with polycystic ovary syndrome: a register-based and population-based cohort study. J Bone Mineral Res.

[43] Richette P, Poitou C, Garnero P, Vicaut E, Bouillot JL, Lacorte JM, Basdevant A, Clement K, Bardin T, Chevalier X (2011). Benefits of massive weight loss on symptoms, systemic inflammation and cartilage turnover in obese patients with knee osteoarthritis. Ann Rheum Dis.

[44] Jungmann PM, Kraus MS, Alizai H, Nardo L, Baum T, Nevitt MC, McCulloch CE, Joseph GB, Lynch JA, Link TM (2013). Association of metabolic risk factors with cartilage degradation assessed by T2 relaxation time at the knee: data from the osteoarthritis initiative. Arthritis Care Res.

[45] Visser AW, de Mutsert R, le Cessie S, den Heijer M, Rosendaal FR, Kloppenburg M (2015). The relative contribution of mechanical stress and systemic processes in different types of osteoarthritis: the NEO study. Ann Rheum Dis.

[46] Glintborg D, Andersen M (2017). Management of endocrine disease: morbidity in polycystic ovary syndrome. Eur J Endocrinol.

[47] Kluzek S, Sanchez-Santos MT, Leyland KM, Judge A, Spector TD, Hart D, Cooper C, Newton J, Arden NK (2015). Painful knee but not hand osteoarthritis is an independent predictor of mortality over 23 years follow-up of a population-based cohort of middle-aged women. Ann Rheum Dis.

[48] Liu Q, Niu J, Li H, Ke Y, Li R, Zhang Y, Lin J (2017). Knee symptomatic osteoarthritis, walking disability, NSAIDs use and all-cause mortality: population-based Wuchuan osteoarthritis study. Sci Rep.

[49] Barbour KE, Lui LY, Nevitt MC, Murphy LB, Helmick CG, Theis KA (2015). Hip osteoarthritis and the risk of all-cause and disease-specific mortality in older women: a population-based cohort study. Arthritis Rheumatol (Hoboken, NJ).

[50] WHO. Healthcare Systems in Transition. Denmark 2002 [Available from: http://www.euro.who.int/__data/assets/pdf_file/0007/98836/DENsum110802.pdf.

[51] Castell MV, van der Pas S, Otero A, Siviero P, Dennison E, Denkinger M, Pedersen N, Sanchez-Martinez M, Queipo R, van Schoor N, Zambon S, Edwards M, Peter R, Schaap L, Deeg D (2015). Osteoarthritis and frailty in elderly individuals across six European countries: results from the European Project on OSteoArthritis (EPOSA). BMC Musculoskelet Disord.

[52] Jorgensen KT, Pedersen BV, Nielsen NM, Hansen AV, Jacobsen S, Frisch M (2011). Socio-demographic factors, reproductive history and risk of osteoarthritis in a cohort of 4.6 million Danish women and men. Osteoarthr Cartil.

[53] Davidsen M, Kjoller M, Helweg-Larsen K (2011). The Danish National Cohort Study (DANCOS). Scand J Public Health.

[54] Wennberg JE, Staiger DO, Sharp SM, Gottlieb DJ, Bevan G, McPherson K, Welch HG (2013). Observational intensity bias associated with illness adjustment: cross sectional analysis of insurance claims. BMJ..

[55] Schisterman EF, Cole SR, Platt RW (2009). Overadjustment bias and unnecessary adjustment in epidemiologic studies. Epidemiology (Cambridge, Mass).

[56] Dashti S, Latiff LA, Zulkefli N, Baharom AB, Minhat HS, Hamid HA, Ismail M, Jafarzadeh Esfehani A, Abu Bakar AS, Binti Sabri NAI (2017). A review on the assessment of the efficacy of common treatments in polycystic ovarian syndrome on prevention of diabetes mellitus. J Family Reprod Health.

