# Supplementary material for: Accelerated osteoarthritis in women with polycystic ovary syndrome: a prospective nationwide registry-based cohort study
Source: Arthritis Res Ther. 2021 Aug 30;23:225. doi: 10.1186/s13075-021-02604-w (PMC8406767; doi:10.1186/s13075-021-02604-w)
Supplement: Supplementary file 1 — Additional file 1: Table S1. ICD-10 codes used for definition of exposure (PCOS), outcomes (hand, hip and knee clinical OA) and exclusion diagnosis. Table S2. Prevalence of knee, hip and hand osteoarthritis in 2015 in Danish population with clinical diagnosis of PCOS and match controls stratified by age groups. Table S3. Prevalence of knee, hip and hand osteoarthritis in 2015 in participants with PCOS, matched controls and combined population group stratified by outcomes (hand, hip and knee clinical OA). Figure S1. Prevalence ratios in 2015 between PCOS and control cohorts for knee, hip and hand osteoarthritis stratified by age groups. Table S4. Incidence rates per 1000 person-year (PY) for knee osteoarthritis by age groups (age at baseline) between patients with and without PCOS. Table S5. Incidence rates per 1000 person-year (PY) for hip osteoarthritis by age groups at baseline between patients with and without PCOS. Table S6. Incidence rates per 1000 person-year (PY) for hand osteoarthritis by age groups at baseline between patients with and without PCOS. [file 13075_2021_2604_MOESM1_ESM.docx]

**Table S1**. ICD-10 codes used for definition of exposure (PCOS), outcomes (hand, hip and knee clinical OA) and exclusion diagnosis.

| **Variable** | **ICD-10 codes** |
| --- | --- |
| Exposure |  |
| PCOS | L68.0 and E28.2 |
| Outcomes |  |
| Knee OA | M17 and M17.0-M17.9 |
| Hip OA | M16 and M16.0-M16.9 |
| Hand OA | M15.1, M15.2, M18 and M18.0-M18.9 |
| Exclusion diagnosis at index date |  |
| Knee OA | M17 and M17.0-M17.9 |
| Hip OA | M16 and M16.0-M16.9 |
| Hand OA | M15.1, M15.2, M18 and M18.0-M18.9 |
| Juvenile arthritis | M08 and M08.0-M08.9 |
| RA | M05, M05.0-M05.9 and M06, M06.0-M06.9 |
| Arthropathic psoriasis | L40.5 |
| Gonococcal or inflammatory spondylopathy | A54.4, M45 and M46.9 |
| Gout | M10 and M10.0-M10.9 |
| Other crystal arthropathies | M11 and M11.0-M11.9 |
| Lupus | M32, M32.0-M32.9 and L93, L93.0-L93.2 |
| Osteogenesis imperfecta | Q78.0 |
| Haemophilia | D66 |
| Ehlers-Danlos syndrome | Q79.6 |

PCOS = Polycystic ovary syndrome; OA = osteoarthritis; RA = rheumatoid arthritis

**Table S2.** Prevalence of knee, hip and hand osteoarthritis in 2015 in Danish population with clinical diagnosis of PCOS and match controls stratified by age groups.

|  | Knee | | Hip | | Hand | |
| --- | --- | --- | --- | --- | --- | --- |
|  | PCOS | Controls | PCOS | Controls | PCOS | Controls |
| *<30 years* | 0.011 | 0.006 | 0.003 | 0.003 | 0.001 | 0.001 |
| *30 to <35 years* | 0.033 | 0.016 | 0.006 | 0.006 | 0.003 | 0.001 |
| *35 to <40 years* | 0.056 | 0.030 | 0.011 | 0.009 | 0.004 | 0.003 |
| *40 to <45 years* | 0.079 | 0.046 | 0.027 | 0.018 | 0.010 | 0.005 |
| *45 to <50 years* | 0.114 | 0.065 | 0.038 | 0.029 | 0.011 | 0.007 |
| *50 to <55 years* | 0.144 | 0.083 | 0.055 | 0.039 | 0.016 | 0.009 |
| *55 to <60 years* | 0.156 | 0.101 | 0.084 | 0.055 | 0.028 | 0.012 |
| ***Overall*** | 0.037 | 0.020 | 0.011 | 0.009 | 0.004 | 0.002 |

PCOS = Polycystic ovary syndrome

**Table S3**. Prevalence of knee, hip and hand osteoarthritis in 2015 in participants with PCOS, matched controls and combined population group stratified by outcomes (hand, hip and knee clinical OA).

| **Side** | **Women with PCOS in 2015 (n=18,844)** | **Matched controls in 2015 (n=56,244)** | **Complete study population in 2015 (n=75,088)** |
| --- | --- | --- | --- |
| Knee osteoarthritis | 3.7% (n=695) | 2.0% (n=1,124) | 2.4% (n=1,819) |
| Hip osteoarthritis | 1.1% (n=211) | 0.9% (n=485) | 0.9% (n=696) |
| Hand osteoarthritis | 0.4% (n=70) | 0.2%(n=108) | 0.2% (n=178) |
| Knee, hip and hand osteoarthritis | 5.2% (n=976) | 3.0% (n=1,717) | 3.6% (n=2,693) |

**Figure S1.** Prevalence ratios in 2015 between PCOS and control cohorts for knee, hip and hand osteoarthritis stratified by age groups.

**Table S4.** Incidence rates per 1000 person-year (PY) for knee osteoarthritis by age groups (age at baseline) between patients with and without PCOS.

|  | **PCOS** | | **Controls** | | Unadjusted  HR* (95% CI) | p-value |
| --- | --- | --- | --- | --- | --- | --- |
| Age | Nº of events | Incidence rate per 1,000 PY (95%CI) | Nº of events | Incidence rate per 1,000 PY (95%CI) |  |  |
| *<30 years* | 97 | 0.9 (0.8; 1.1) | 153 | 0.5 (0.4; 0.6) | 1.9 (1.5; 2.5) | <0.001 |
| *30 to <35 years* | 108 | 2.5 (2.1; 3.1) | 158 | 1.2 (1.1; 1.5) | 2.0 (1.6; 2.6) | <0.001 |
| *35 to <40 years* | 120 | 4.0 (3.4; 4.8) | 201 | 2.3 (2.0; 2.6) | 1.8 (1.4; 2.3) | <0.001 |
| *40 to <45 years* | 85 | 5.2 (4.2; 6.4) | 150 | 3.0 (2.6; 3.6) | 1.8 (1.3; 2.3) | <0.001 |
| *45 to <50 years* | 85 | 8.3 (6.7; 10.2) | 127 | 4.1 (3.5; 4.9) | 2.1 (1.6; 2.7) | <0.001 |
| *50 to <55 years* | 56 | 8.9 (6.8; 11.6) | 89 | 4.8 (3.9; 5.9) | 2.1 (1.5; 2.7) | <0.001 |
| *55 to <60 years* | 37 | 9.3 (6.7; 12.8) | 73 | 6.2 (4.9; 7.8) | 1.5 (1.0; 2.3) | 0.056 |
| *Total* | 588 | 2.8 (2.5; 3.0) | 951 | 1.5 (1.4; 1.6) | 1.9 (1.7;2.1) | <0.001 |
| Sensitivity analysis (after excluding individual with documented obesity) | | | | | | |
| *Total* | 357 | 2.3 (2.1; 2,5) | 825 | 1.4 (1.4; 1.5) | 1.6 (1.4;1.8) | <0.001 |

*Reference = control group

PCOS = Polycystic ovary syndrome; HR = Hazard Ratio

**Table S5**. Incidence rates per 1000 person-year (PY) for hip osteoarthritis by age groups at baseline between patients with and without PCOS.

|  | **PCOS** | | **Controls** | | Unadjusted  HR* (95% CI) | p-value |
| --- | --- | --- | --- | --- | --- | --- |
| Age | Nº of events | Incidence rate per 1000 PY (95%CI) | Nº of events | Incidence rate per 1000 PY (95%CI) |  |  |
| *<30 years* | 26 | 0.2 (0.2; 0.4) | 72 | 0.2 (0.2; 0.3) | 1.1 (0.7; 1.7) | 0.666 |
| *30 to <35 years* | 16 | 0.4 (0.2; 0.6) | 53 | 0.4 (0.3; 0.5) | 0.9 (0.5; 1.6) | 0.745 |
| *35 to <40 years* | 25 | 0.8 (0.6; 1.2) | 58 | 0.6 (0.5; 0.8) | 1.3 (0.8; 2.0) | 0.346 |
| *40 to <45 years* | 28 | 1.7 (1.2; 2.4) | 66 | 1.3 (1.0; 1.7) | 1.4 (0.9; 2.1) | 0.193 |
| *45 to <50 years* | 28 | 2.6 (1.8; 3.8) | 54 | 1.7 (1.3; 2.3) | 1.4 (0.9; 2.3) | 0.129 |
| *50 to <55 years* | 25 | 3.8 (2.5; 5.6) | 48 | 2.5(1.9; 3.4) | 1.4 (0.9; 2.3) | 0.150 |
| *55 to <60 years* | 25 | 6.1 (4.1; 9.0) | 39 | 3.2 (2.4; 4.4) | 1.8 (1,1;3.1) | 0.023 |
| *Total* | 173 | 0.8 (0.7; 0.9) | 390 | 0.6 (0.6; 0.7) | 1.3 (1.1; 1.6) | 0.004 |
| Sensitivity analysis (after excluding individual with documented obesity) | | | | | | |
| *Total* | 125 | 0.8 (0.7; 0.9) | 352 | 0.6 (0.5; 0.6) | 1.2 (1.0; 1.5) | 0.066 |

*Reference = control group

PCOS = Polycystic ovary syndrome; HR = Hazard Ratio

**Table S6.** Incidence rates per 1000 person-year (PY) for hand osteoarthritis by age groups at baseline between patients with and without PCOS

|  | **PCOS** | | **Controls** | | Unadjusted  HR* (95% CI) | p-value |
| --- | --- | --- | --- | --- | --- | --- |
| Age | Nº of events | Incidence rate per 1000 PY (95%CI) | Nº of events | Incidence rate per 1000 PY (95%CI) |  |  |
| *<30 years* | 7 | 0.1 (0.0; 0.1) | 14 | 0.0 (0.0; 0.1) | 1.6 (0.6; 4.0) | 0.329 |
| *30 to <35 years* | 9 | 0.2 (0.1; 0.4) | 9 | 0.1 (0.0; 0.1) | 3.0 (1.2; 7.6) | 0.020 |
| *35 to <40 years* | 10 | 0.3 (0.2; 0.6) | 20 | 0.2 (0.1; 0.3) | 1.4 (0.7; 3.1) | 0.350 |
| *40 to <45 years* | 9 | 0.5 (0.3; 1.0) | 15 | 0.3 (0.2; 0.5) | 1.7 (0.8; 4.2) | 0.173 |
| *45 to <50 years* | 7 | 0.6 (0.3; 1.4) | 20 | 0.6 (0.4; 1.0) | 1.1 (0.5; 2.7) | 0.819 |
| *50 to <55 years* | 11 | 1.7 (0.9; 3.0) | 10 | 0.5 (0.3; 1.0) | 3.9 (1.6; 9.7) | 0.004 |
| *55 to <60 years* | 2 | 0.5 (0.1; 1.9) | 8 | 0.7 (0.3; 1.3) | 0.7 (0.1; 3.2) | 0.635 |
| *Total* | 55 | 0.3 (0.2; 0.3) | 96 | 0.1 (0.1; 0.2) | 1.8 (1.3; 2.4) | 0.001 |
| Sensitivity analysis (after excluding individual with documented obesity) | | | | | | |
| *Total* | 40 | 0.3 (0.2; 0.3) | 91 | 0.2 (0.1; 0.2) | 1.6 (1.1; 2.3) | 0.026 |

*Reference = control group

PCOS = Polycystic ovary syndrome; HR = Hazard Ratio
